# Interaction of microRNA-21/145 and Smad3 domain-specific phosphorylation in hepatocellular carcinoma

**DOI:** 10.18632/oncotarget.17709

**Published:** 2017-05-09

**Authors:** Ji Yu Wang, Meng Fang, Alex Boye, Chao Wu, Jia Jun Wu, Ying Ma, Shu Hou, Yue Kan, Yan Yang

**Affiliations:** ^1^ Department of Pharmacology and Institute of Natural Medicine, Anhui Medical University, Hefei 230032, China

**Keywords:** hepatocellular carcinoma, microRNA-21, microRNA-145, pSmad3C, pSmad3L

## Abstract

MicroRNAs 21 and 145 exhibit inverse expression in Hepatocellular carcinoma (HCC), but how they relate to Smad3 C-terminal and Link region phosphorylation (pSmad3C and pSmad3L) downstream of TGF-β/MAPK signaling, remains inconclusive. Our results suggest microRNA-145 targets Smad3 in HepG2 cells. Decreased tumor volume and increased apoptosis were produced in both microRNA-21 antagomir and microRNA-145 agomir groups compared to controls. Inhibition of TβRI and MAPK (ERK, JNK, and p38) activation respectively produced decreased microRNA-21 but increased microRNA-145 expression. Correspondingly, the expression level of pSmad3C obviously increased while pSmad3L decreased in microRNA-145 agomir-group and the expression of pSmad3C/3L were not markedly changed but pERK, pJNK, pp38 decreased in microRNA-21 antagomir-group compared to controls. On the other hand, microRNA-145 and 21 increased respectively in xenografts of HepG2 cells transfected with Smad3 EPSM and 3S-A plasmid, and this correlated with the overexpression of pSmad3C and pSmad3L respectively compared to control. To conclude, microRNA-21 promotes tumor progression in a MAPK-dependent manner while microRNA-145 suppresses it via domain-specific phosphorylation of Smad3 in HCC. Meanwhile, increased pSmad3C/3L lead to the up-regulation of microRNA-145/21 respectively. The interaction between pSmad3C/3L and microRNA-145/21 regulates HCC progression and the switch of pSmad3C/3L may serve as an important target for HCC therapy.

## INTRODUCTION

Hepatocellular carcinoma (HCC) is one of the most common malignancies worldwide. Despite recent FDA approval of sorafenib for the treatment of advanced HCC, treatment of HCC remains a major challenge. This culminates from complexity of many dysregulated cell signaling pathways implicated in HCC, including but not limited to transforming growth factor-beta (TGF-β) and mitogen-activated protein kinase (MAPK) signaling pathways, which interact directly or indirectly with microRNAs that play crucial roles in carcinogenesis. Notably, TGF-β signal mediated through MAPK-dependent Linker phosphorylation of Smad2/3 tend to promote fibrogenesis and oncogenesis, while pSmad3C-mediated TGF-β signaling produce tumor suppressive effects, as a result modulation of phospho-domains of receptor-mediated Smads has become a crucial target for therapeutic exploration. As a common denominator to MAPK-dependent (Smad2L and Smad3L) and MAPK-independent (Smad2C and Smad3C) phosphorylation of Smad2/3 downstream of TGF-β signal is the critical role of TGF-β type 1 (TβRI) and 2 (TβRII) receptors, which are ligand-activated to initiate TGF-β signaling cascade. Receptor-mediated Smads (Smad2 and Smad3 specific for TGF-β) as well as common Smad (Smad4) play crucial roles for the transduction of TGF-β signal from its receptors to the nucleus [1]. Hetero-complex formation between Smad2/3/4 aided by both nuclear pore and importin proteins ensure nuclear relocation as well as cyto-nuclear shuttling of the Smad proteins in quick response to TGF-β receptor activity [2], though Smad4 is dispensable for the nuclear translocation of phosphorylated Smad2/3 in a MAPK-dependent manner [3]. In the nucleus, the Smads bind to the Smad binding elements (SBE) in the promoter of TGF-β target genes just as activated MAPKs (ERK, JNK, and p38) bind to their specific sites as well as interact with many transcription factors to regulate target gene transcription. Generally but not universally, post-transcriptional output of target genes including that of TGF-β targets, mostly oncogenes are regulated at multi-level by many microRNAs through repression or activation of the 3’-UTR of target mRNAs [4, 5]. Mis-expression of specific microRNAs correlates with the progression of some cancer subtypes [6]. In view of this, microRNAs elevated and those decreased in specific cancers are designated as oncogenes and tumor suppressors respectively [6, 7]. Functionally, microRNAs interact at multi-level with oncogenes and tumor suppressor gene networks to initiate most human cancers [7, 8]. For example, microRNA-21 and microRNA-145 were reported up-regulated and down-regulated respectively in HCC and this pattern of expression correlated with increase in a number of oncogenes but a decrease in tumor suppressor genes respectively [9-12]. Four computational methods (Miranda, TargetScan, PicTar and RNA22) and a number of reports have all predicted programmed cell death protein 4 (PDCD4), a neoplastic transformation inhibitor in both humans and mouse [13-18], as well as inhibitory Smad7 [19, 20], PTEN [12], Smad2 [21], and TGF-β receptors [17] to be confirmed targets of miR-21. On the other hand, miR-145 is decreased in HCC [22, 23] and this correlated with a decrease in its tentative targets [10, 23]. Putatively, MAP3K, MAP4K4, MYCN, FOS, YES, FLI-1 [22, 24, 25], IRS1, IRS2, Oct4 [23], and Smad3 [26] are predicted targets of microRNA-145. Of all the predicted targets of microRNA-145, Smad3 is integral in view of its role in versatile TGF-β diverse signaling networks in both homeostasis and disease pathogenesis such as cancer.

TGF-β expression is up-regulated in tumor cells where it promotes metastatic phenotype [27]. TGF-β and MAPK mediated phosphorylation of Smad2 at the Linker and C-terminal respectively was implicated in liver fibrosis [28], and constitutive Smad3 phosphorylation in myofibroblast [29, 30]. pSmad3L but not pSmad3C was reported up-regulated in hepato-carcinogenesis [31]. Autocrine TGF-β secretion increases basal microRNA-21 expression in tumor cells [32]. Meanwhile, microRNA-21 interacts with downstream mediators of TGF-β (Smad2 and Smad3) at multi-level to post-transcriptionally regulate up-regulation of oncogene but down-regulation of tumor suppressor genes (PDCD4 and PTEN) [32] to promote carcinogenesis [33]. On the other hand, microRNA-145 modulates Smad3 in many fibrogenic disorders [26, 34]. Functionally, the specific site or phospho-domain of Smad3 targeted is indispensable, in view of the fact that domain-specific phosphorylation of Smad3 play different roles in hepato-carcinogenesis coupled with the fact that inverse expression of microRNA-21 and microRNA-145 have far reaching implications for tumor progression. We strongly suspect that inverse microRNA-21 and microRNA-145 expression may be related to Smad3 phosphorylation switch at Linker and the C-terminal to produce opposing modulatory effects in hepato-carcinogenesis. On this basis, we hypothesize that inverse expression of microRNA-21 and microRNA-145 in hepato-carcinogenesis may switch domain-specific phosphorylation of Smad3 in a TβRI/MAPK-dependent manner. To test this hypothesis, first we investigated effect of microRNA-145 agomir on Smad3 3’-UTR. Subsequently, xenograft models of HCC involving male BALB/c nude mice and humanized hepatoma cell lines (HepG2 cells) were used to investigate how microRNA-21 antagomir and microRNA-145 agomir mediate tumor progression and apoptosis and the underlying mechanisms involving domain-specific Smad3 phosphorylation mediated by TGF-β and MAPK signaling.

## RESULTS

### MicroRNA-145 targets SMAD3 3’-UTR (1397–1404) seed site in HepG2 cells

**Figure 1:**
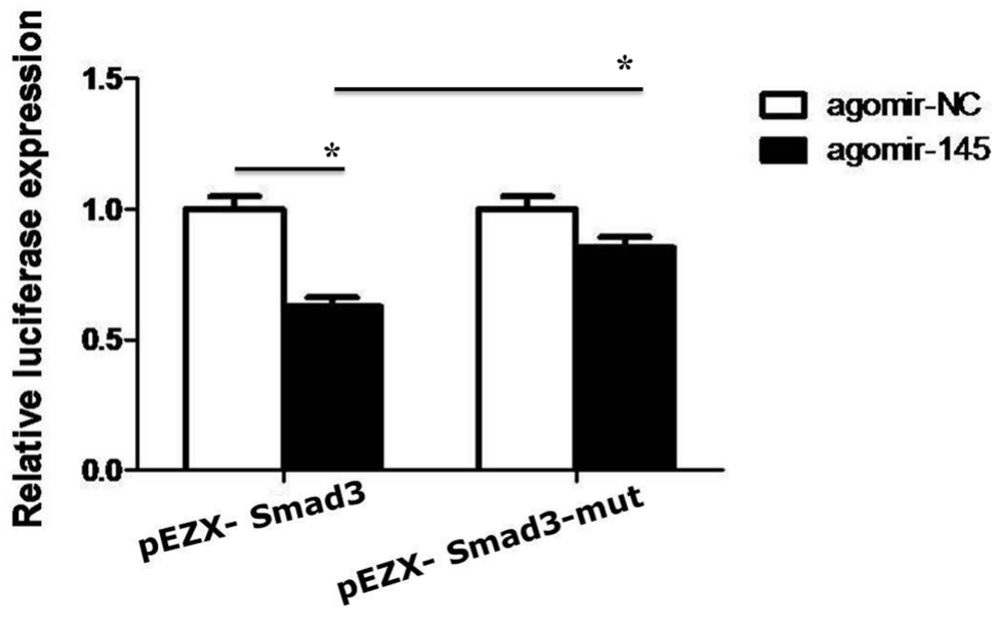
The luciferase activity was measured in HepG2 cells co-transfected with miR-145 agomir or negative control and Smad3 3’-UTR wild-type, mutant vector for 48h ^*^P <0.05.

MicroRNAs post-transcriptionally regulate the expression of target genes through complementary pairing to the 3’-UTR of their target mRNA [32, 35]. In view of this it was necessary to identify the specific site on Smad3 3’-UTR regulated by microRNA-145, giving that microRNA-145 is reported to have down-regulated Smad3 in some cell types [26, 36]. From analysis of microRNA target database using computational methods and supported by earlier studies two seed sites in the Smad3 3’-UTR were found to be target sites for microRNA-145 [37]. Out of the two sites, one was functional and encodes genes implicated in tumor progression and inflammatory related disorders [37]. Interestingly, using luciferase reporter construct carrying the Smad3 3’-UTR (pEZX-Smad3) with the functional seed site, co-transfection of HepG2 cells with miR-145 agomir, produced decreased luciferase activity (Figure 1). Moreover, this inhibitory effect was attenuated by the 1397-1404 Seed Site mutation (pEZX-Smad3-mut) (Figure 1). These data suggest that microRNA-145 targets Smad3 in HepG2 cells.

### Down-regulated microRNA-21 and up-regulated microRNA-145 expressions produced opposing effects on tumor burden and promoting effects on apoptosis *in vivo*

**Figure 2:**
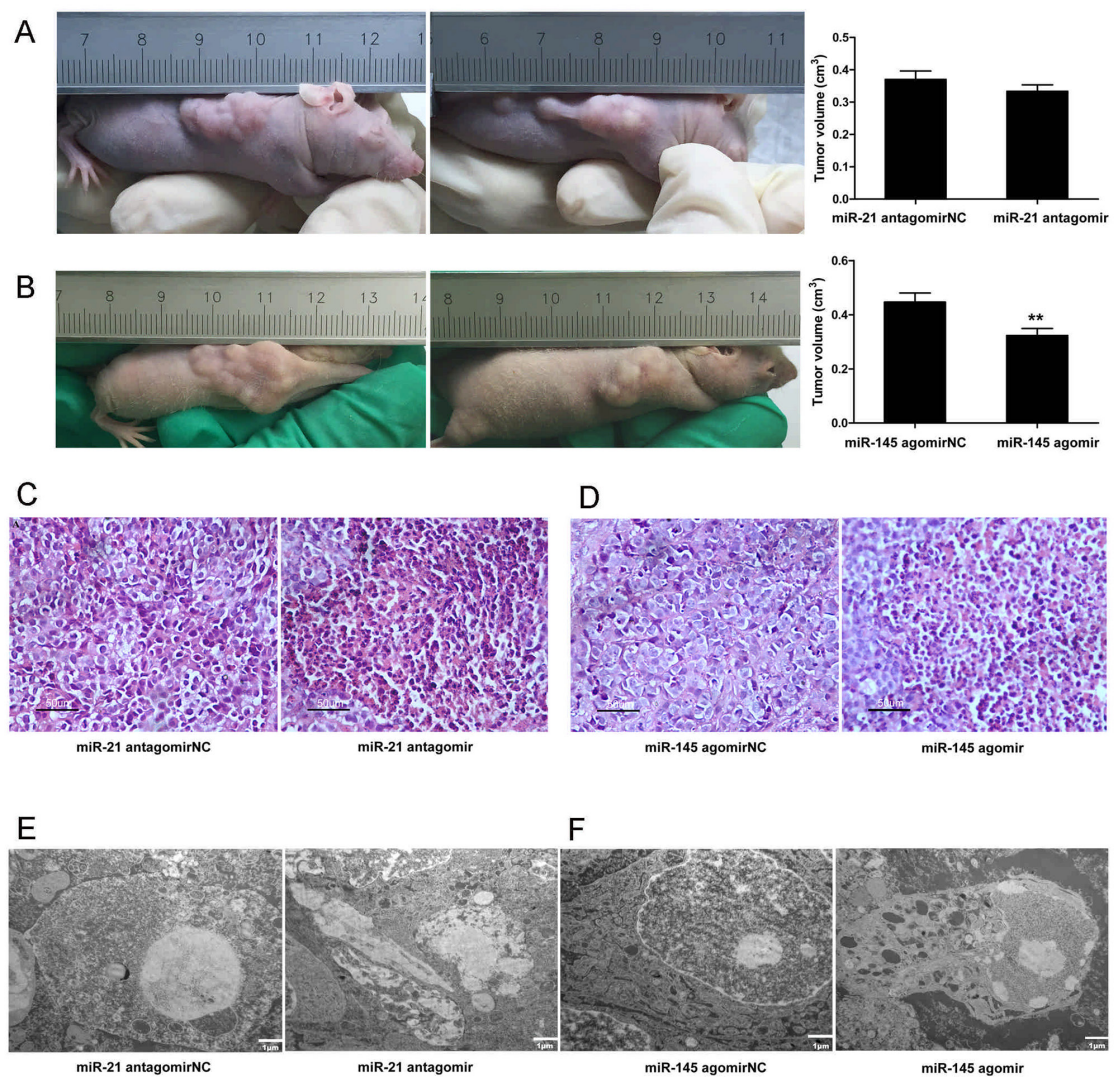
Down-regulated microRNA-21 and up-regulated microRNA-145 expressions produced opposing effects on tumor burden and promoting effects on apoptosis *in vivo* **(A)**Tumor volumes of injected microRNA-21 antagomir were measured at the end of the experiment (28 days after tumor formation). **(B)** Tumor volumes of injected microRNA-145 agomir were measured at the end of the experiment (28 days after tumor formation). ^**^P <0.01. **(C)** Cell morphological changes in xenograft tumors of injected microRNA-21 antagomir were measured with Hematoxylin Eosin staining, ×400, Scale bar, 50µm. **(D)** Cell morphological changes in tumors of injected microRNA-145 agomir were measured with Hematoxylin Eosin staining, ×400, Scale bar, 50µm. **(E)** Cell apoptosis in tumors of injected microRNA-21 antagomir were measured with Electron microscope. ×6000, Scale bar, 1µm. **(F)** Cell apoptosis in tumors of injected microRNA-145 agomir were measured with Electron microscope, ×6000, Scale bar, 1µm; ×15000, Scale bar, 500nm.

Tumor burden and tumor cell survival are two important phenotypic hallmarks of cancer, and they are related to dysregulated TGF-β signaling [38]. Tumor transplantation model of cancer involving xenogeneic tumor cells can be achieved in immuno-compromised animals such as nude mice [39]. Such cancer models allow for the study and investigation of cancer phenotypic hallmarks and the underlying dysregulated cell signaling and molecular pathways. To study the involvement of microRNA-21 and 145 in tumor burden and survival in HCC, transplanted HepG2 cell tumors in nude mice were treated with microRNA-21 antagomir and microRNA-145 agomir respectively as against their respective negative controls. Notably, tumor volume was decreased in xenograft tumors treated with microRNA-21 antagomir and microRNA-145 agomir compared to those tumors that received microRNA-21 antagomirNC and microRNA-145 agomirNC treatments (Figure 2A and 2B). Pathologically, there was a obvious nucleus shrivel in the microRNA-21 antagomir and microRNA-145 agomir group compared to antagomirNC and agomirNC group (Figure 2C and 2D). And this patho-morphological change correlated with promoting effects on apoptosis (Figure 2E and 2F). These results preliminarily suggest that down-regulated microR-21 or up-regulated microR-145 plays an important role in inhibiting HCC growth and accelerating the apoptosis.

### Down-regulated microRNA-21 expression suppressed MAPK pathway and up-regulated microRNA-145 expression switched Smad3 phosphorylation at Linker and C-terminal in HCC

**Figure 3:**
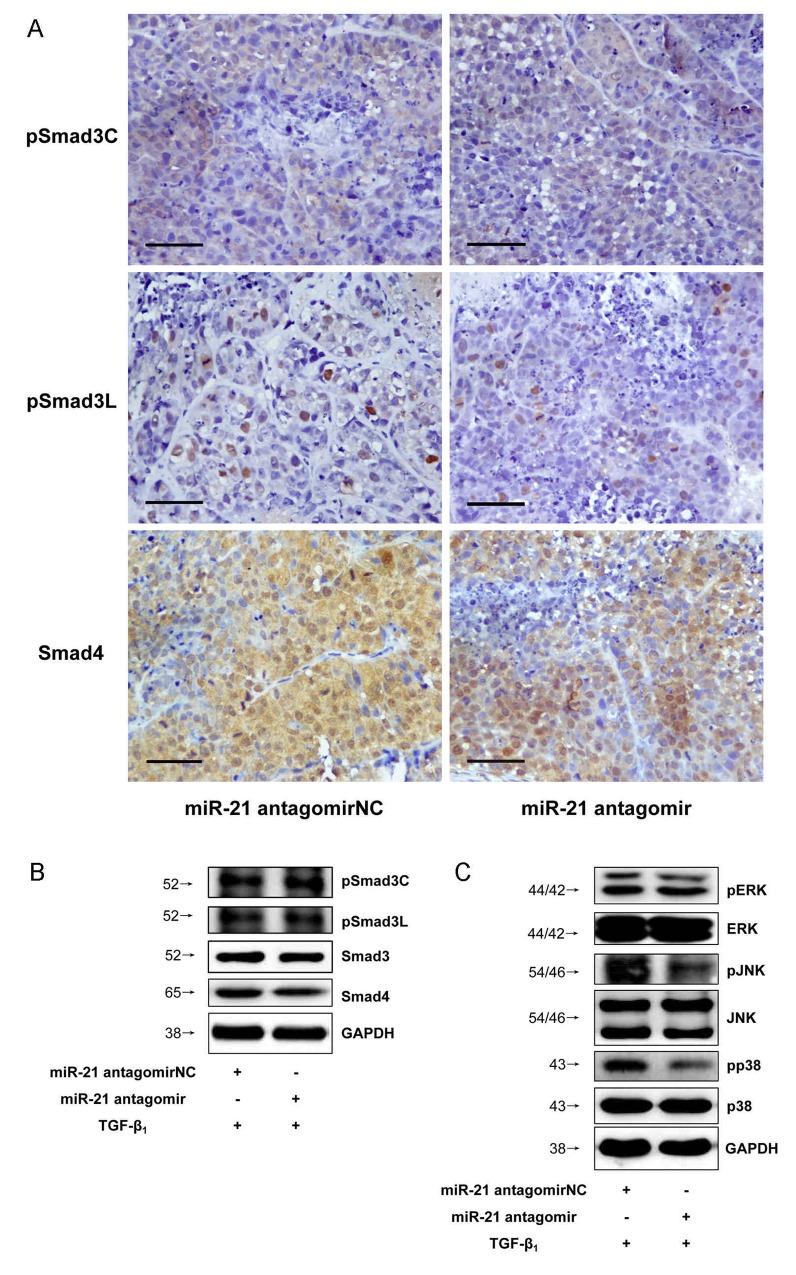
Down-regulated microRNA-21 expression suppressed MAPK pathway in HCC **(A)**PSmad3C and pSmad3L expressions in xenograft tumors of injected microRNA-21 antagomir were measured with Immunohistochemistry, ×400, Scale bar, 50µm. **(B)** PSmad3C and pSmad3L expressions in HepG2 cells transfected with microRNA-21 antagomir were measured with Western-blot. **(C)** PERK1/2, pJNK and pp38 expressions in HepG2 cells transfected with microRNA-21 antagomir were measured with Western-blot.

**Figure 4:**
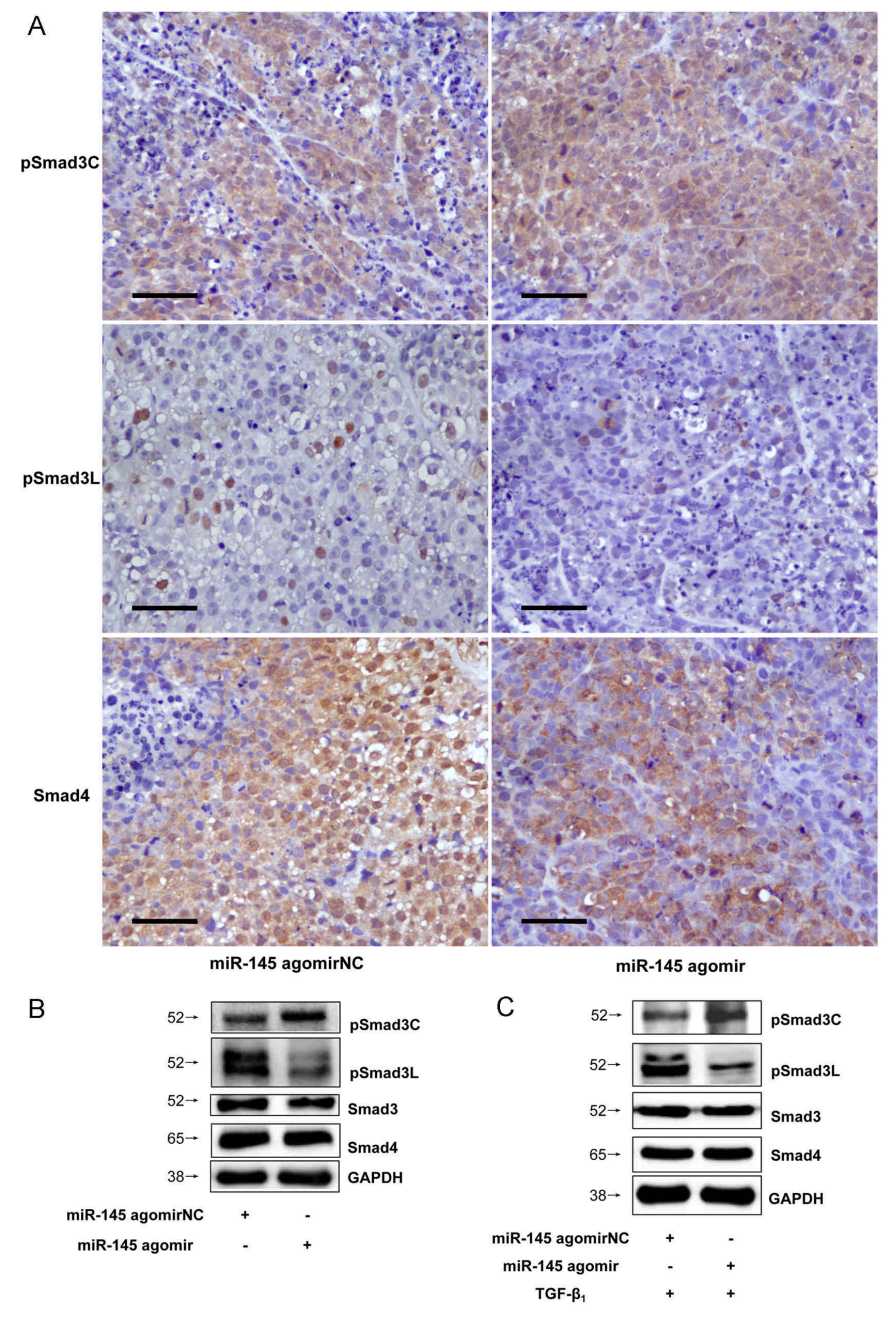
Up-regulated microRNA-145 expression switched Smad3 phosphorylation at Linker and C-terminal in HCC **(A)**PSmad3C and pSmad3L expressions in tumors of injected microRNA-145 agomir were measured with Immunohistochemistry, ×400, Scale bar, 50µm. **(B)** PSmad3C and pSmad3L expressions in tumors of injected microRNA-145 agomir were measured with Western-blot. **(C)** PSmad3C and pSmad3L expressions in HepG2 cells transfected with microRNA-145 agomir were measured with Western-blot.

MicroRNA-21 and microRNA-145 inverse expression pattern in HCC was found to be related to MAPK and TβRI (Figure 8). In view of the roles that MAPK and TβRI play with respect to Smad3L and Smad3C phosphorylation respectively, we investigated whether the regulation of MAPK and TβRI by microRNA-21 and 145 are directly related to Smad3 phosphorylation. However there were not obvious changes in the expression of pSmad3C and pSmad3L in the HepG2 cells transfected with miR-21 antagomir compared with antagomirNC-group (Figure 3A and 3B). We futher explore the effects of decreased microRNA-21 on MAPK pathway, the expression of pERK1/2, pJNK1/2 and pp38 were decreased in microRNA-21 antagomir-group compared to antagomirNC-group (Figure 3C). The results indicate that down-regulated miR-21 expression can suppress the activation of MAPK signaling pathway in HepG2 cells. Of note, the expression level of pSmad3C was up-regulated in miR-145 agomir-group and pSmad3L expression level was obviously decreased compared with agomirNC-group *in vitro* and *in vivo* (Figures 4A, 3B and 3C). These data indicate that microRNA-145 switchs pSmad3L to pSmad3C to suppress tumor progression in HCC.

**Figure 5:**
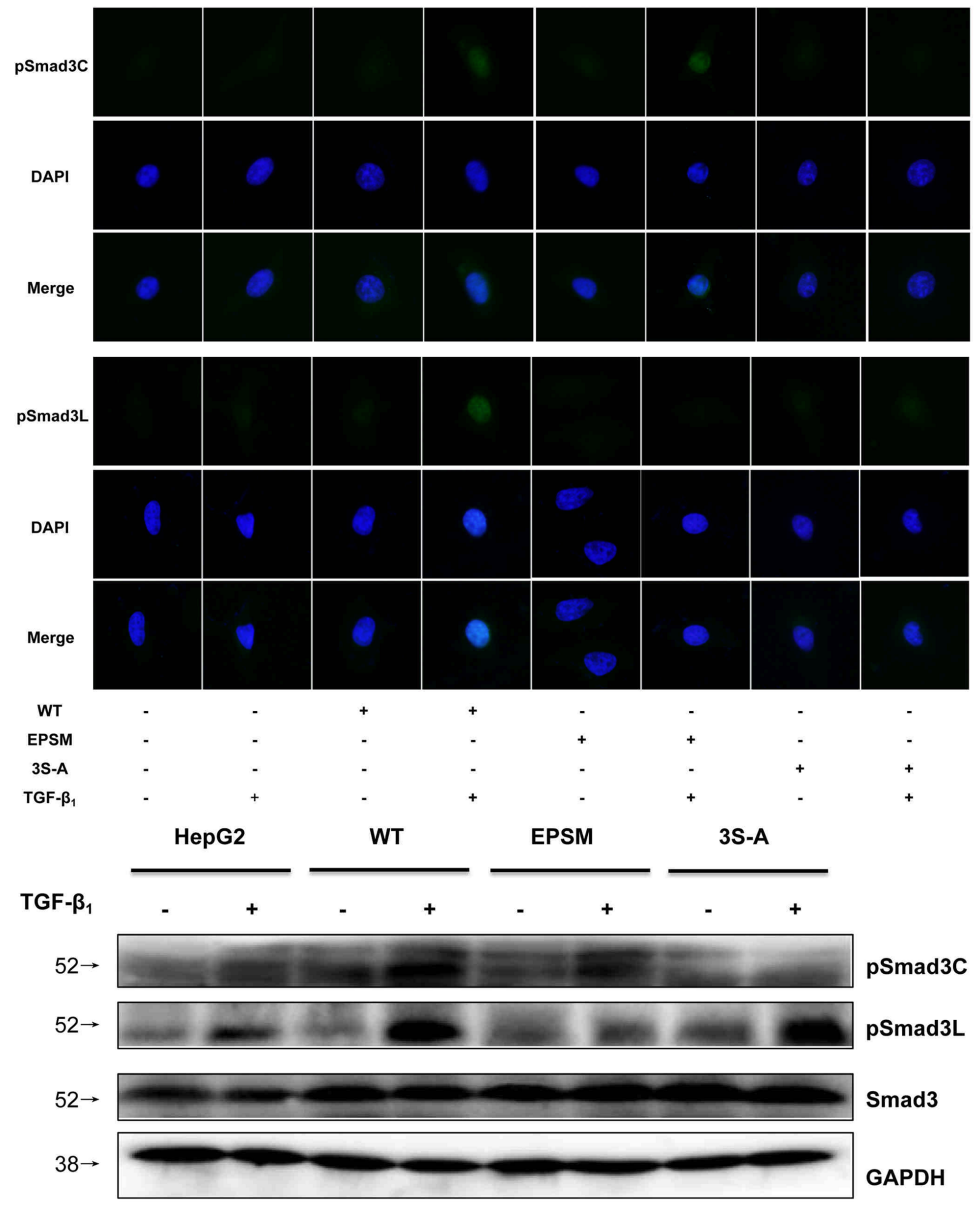
HepG2 cells were transfected with Smad3 WT, Smad3 EPSM, Smad3 3S-A The transfection efficiency were confirmed by immunofluorescence and Western-blot.

**Figure 6:**
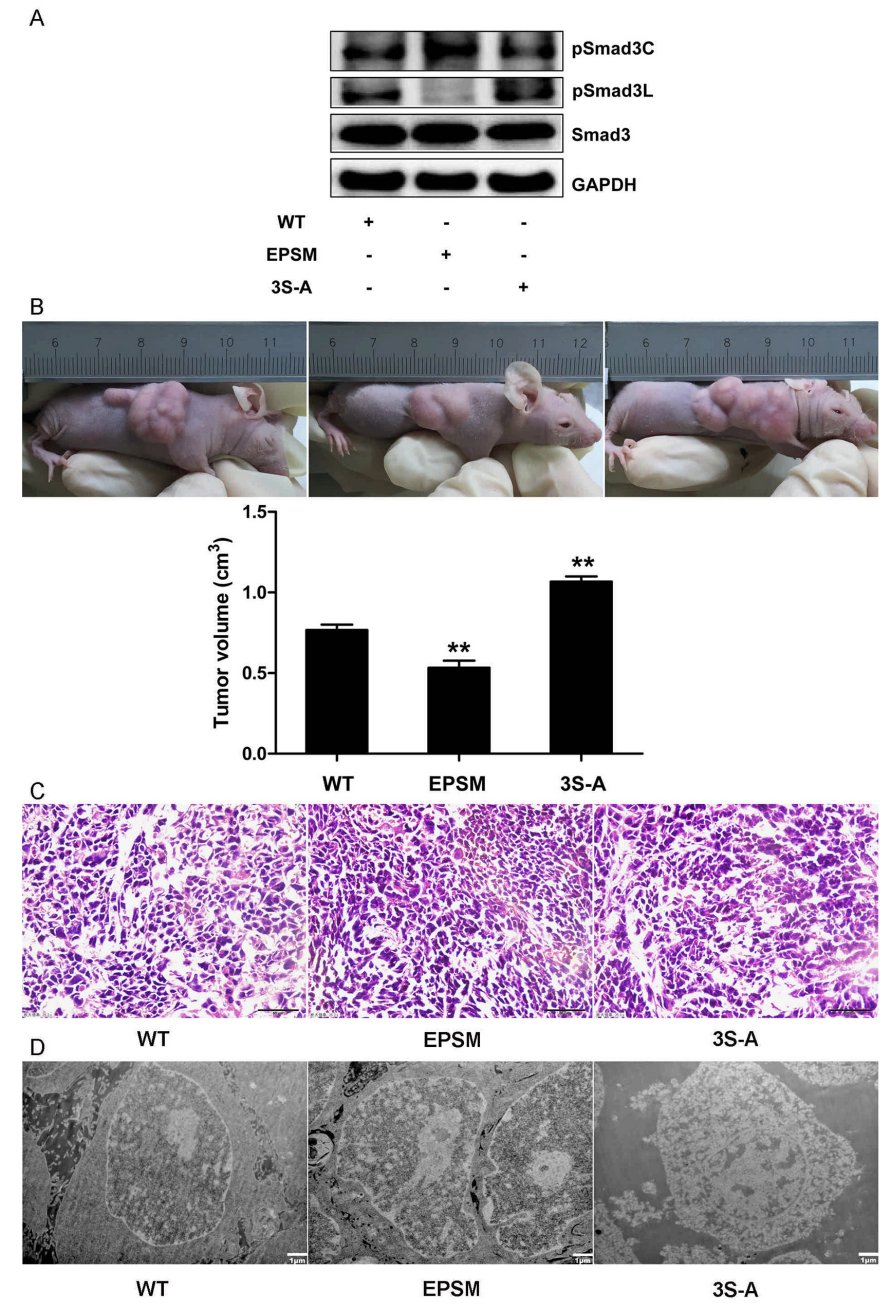
Smad3 phosphorylation at Linker and C-terminal effects on tumor progression in HCC **(A)**HepG2 cells infected with Smad3 WT, EPSM, 3S-A were injected subcateously into nude mice. Western-blot analysis of pSmad3C/3L expression in xenograft tumors. **(B)** Tumor volumes were measured at the end of the experiment (28 days after tumor formation). ^**^P <0.01. **(C)** Cell morphological changes in tumors were measured with Hematoxylin Eosin staining, ×400, Scale bar, 50µm. **(D)** Cell apoptosis in tumors were measured with Electron microscope, ×5000, Scale bar, 1µm.

**Figure 7:**
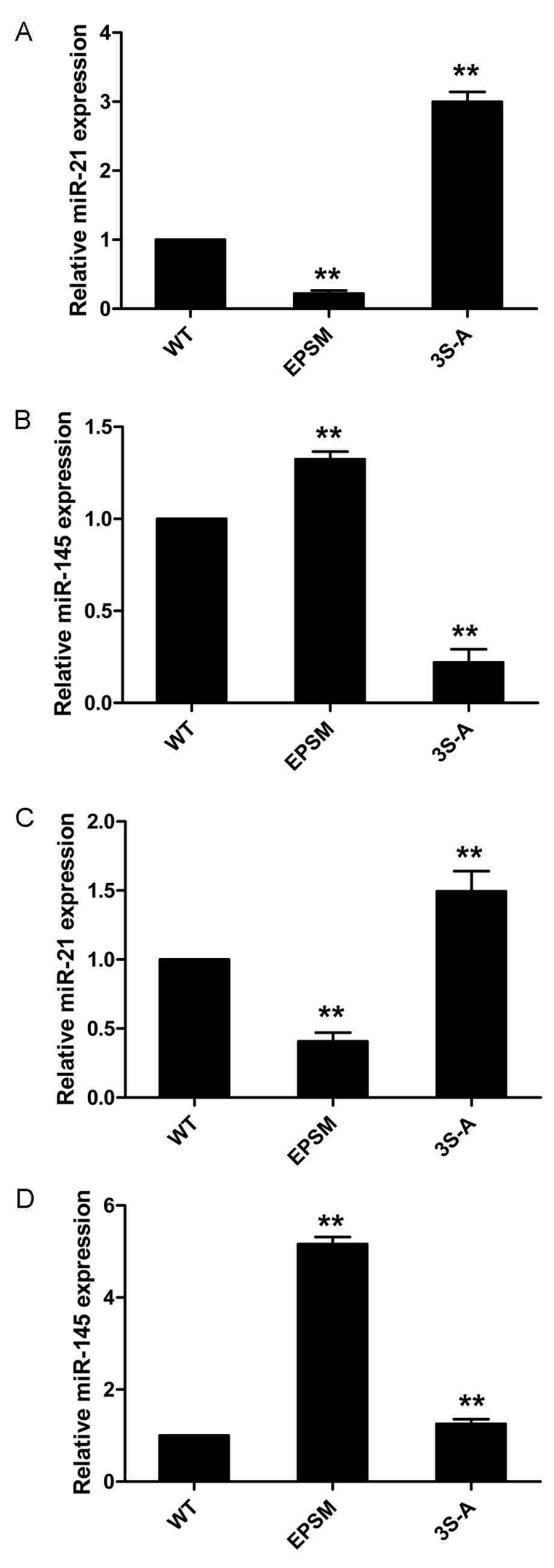
Smad3 phosphorylation at Linker and C-terminal effects on microRNA-21 and 145 expressions in HCC **(A)**HepG2 cells transfected with Smad3 WT, EPSM, 3S-A were injected subcutaneously into nude mice. MicroRNA-21 expression were measured with qRT-PCR analysis. **(B)** MicroRNA-145 expression were measured with qRT-PCR analysis. **(C)** MicroRNA-21 expression in HepG2 cells transfected with Smad3 WT, EPSM, 3S-A were measured with qRT-PCR analysis. **(D)** MicroRNA-145 expression in HepG2 cells transfected with Smad3 WT, EPSM, 3S-A were measured with qRT-PCR analysis. ^**^P <0.01.

**Figure 8:**
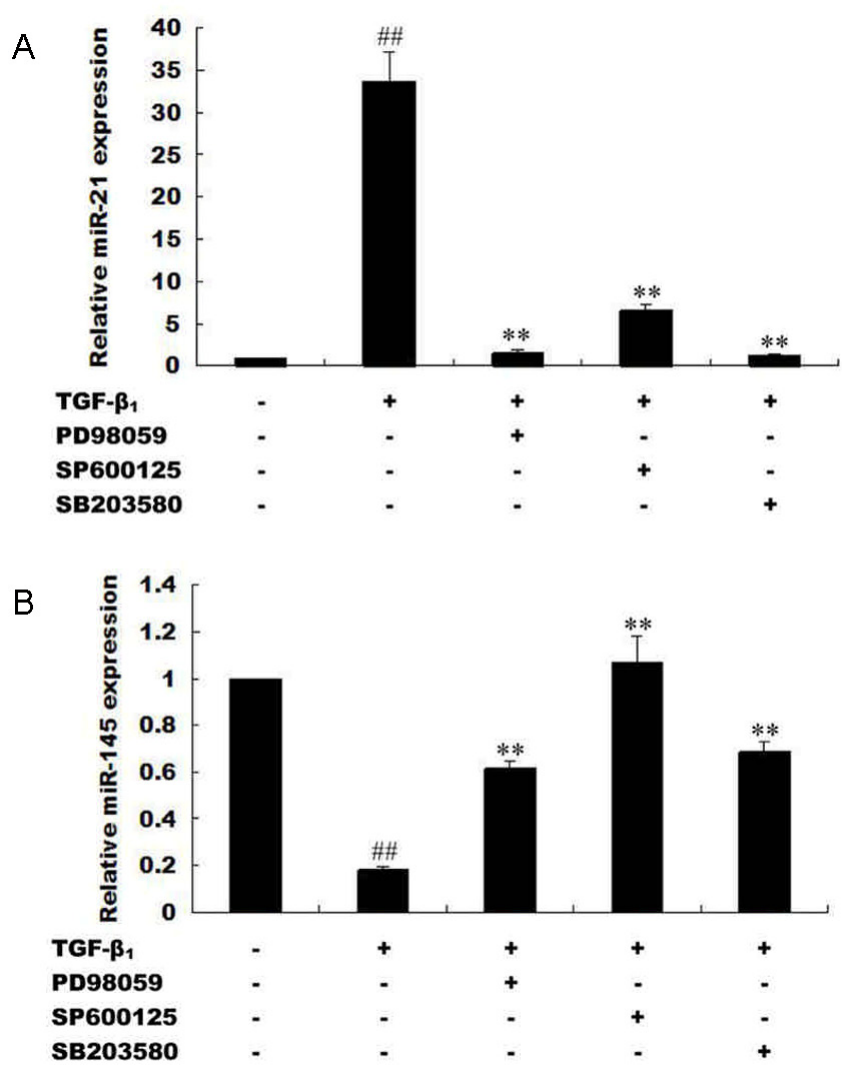
Expression of microRNA-21 and microRNA-145 is mediated by MAPK and TβRI activation **(A)**HepG2 cells were processed with TβRI-specific inhibitor (SB431542) and stimulated with exogenous TGF-β_1_. MicroRNA-21 expression were measured with qRT-PCR. **(B)** HepG2 cells were processed with MAPK-specific inhibitors (PD98059, SP600125, SB203580) and stimulated with exogenous TGF-β_1_. MicroRNA-21 expression were measured with qRT-PCR. **(C)** HepG2 cells were processed with TβRI-specific inhibitor (SB431542) and stimulated with exogenous TGF-β_1_. MicroRNA-145 expression were measured with qRT-PCR. **(D)** HepG2 cells were processed with MAPK-specific inhibitors (PD98059, SP600125, SB203580) and stimulated with exogenous TGF-β_1_. MicroRNA-145 expression were measured with qRT-PCR. ^**^P <0.01, compared with control group without TGF-β_1_ stimulation; ^##^*P* <0.01, compared with TGF-β_1_ stimulated group.

**Figure:**
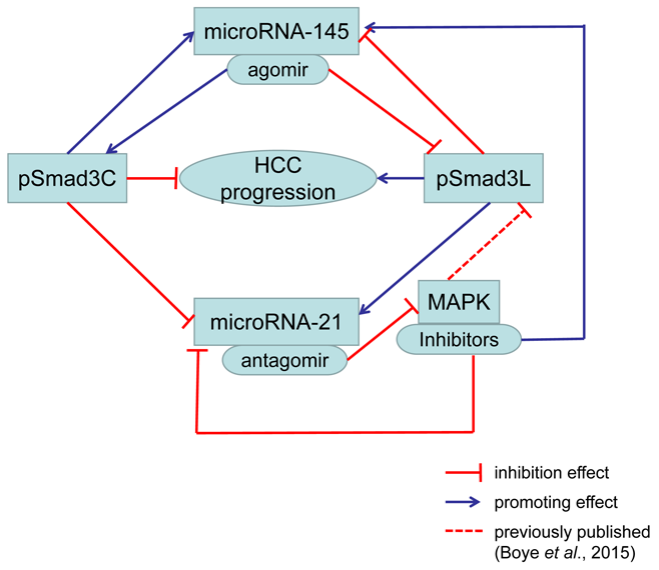
Graphical abstract: Increased pSmadC promoted miR-145 and inhibited miR-21 Increased pSmad3L inhibited miR-145 and promoted miR-21. Up-regulated miR-145 increased pSmad3C and decreased pSmad3L. Down-regulated miR-21 suppressed MAPK activation. MAPK inhibitors induced decreased miR-21, pSmad3L and increased miR-145. Above all, increased pSmad3C and decreased pSmad3L suppressed HCC progression.

### Smad3 phosphorylation at Linker and C-terminal effects on microRNA-21 and 145 expressions in HCC

Successful transfection of HepG2 cells with Smad3-WT, Smad3 EPSM, and Smad3 3S-A respectively was confirmed (Figure 5). Then, sub-cutaneous injection of nude mice with HepG2 cells successfully transfected with Smad3 WT, Smad3 EPSM, and Smad3 3S-A respectively, pSmad3C was up-regulated in EPSM-tumors and pSmad3L was up-regulated in 3S-A-tumors compared to WT-tumors (Figure 6A). Tumor volume of EPSM-group was significantly reduced (*P*<0.01) compared with WT-group. On the contrary, the tumor volume in 3S-A-group were indeed increased (*P*<0.01) compared with WT-group (Figure 6B). Nuclear condensation and shrinkage were observed in EPSM-group compared to WT-group (Figure 6C). And this correlated with increased tumor cell apoptosis in EPSM-tumors compared to WT-tumors (Figure 6D). In contrast to these observations, tumor cells grew well in 3S-A-group compared to WT-group. And these observations correspondingly led to elevated expression of microRNA-145 and 21 respectively in 3S-A-group and EPSM-group compared to their respective expressions in WT-group *in vivo* and *in vitro* (Figure 7). These results suggest that increased pSmad3C or decreased pSmad3L expression reduce tumor-burden and promotes apoptosis. And Smad3 domain-specific phosphorylation interacts with the expressions of microRNA-145 and 21 in HCC.

### Expression of microRNA-21 and microRNA-145 is mediated by MAPK and TβRI activation

Phosphorylation of Smad3 at the Linker and C-terminal are mediated by MAPK (ERK, JNK, and p38) and TβRI respectively [31, 40]. Direct or indirect TGF-β activation of the MAPK pathway leads to phosphorylation of Smad2/3 at the Linker region, while trans-phosphorylation of TβRI by TβRII secondary to TGF-β stimulation leads to phosphorylation of Smad2/3 at the C-terminal [30, 31]. Linker phosphorylated Smad2/3 promotes oncogenesis, while C-terminal phosphorylation of Smad3 tend to produce tumor suppression [40]. In view of this, we sought to find out whether; the inverse expressions of microRNA-21 and microRNA-145 are related to TGF-β stimulation of MAPK and TβRI. Interestingly, blockage of MAPK pathway and TβRI by using MAPK-specific inhibitors (PD98059, SP600125, SB203580) and TβRI-specific inhibitor (SB431542) in exogenous TGF-β_1_-stimulated HepG2 cells produced decreased microRNA-21 fold change but increased that of microRNA-145 (Figure 8). These results suggest that MAPK pathway activation induced increased microRNA-21 and decreased microRNA-145 expression and futher influence the progression of hepatocellular carcinoma.

## DISCUSSION

We demonstrate that Smad3 phosphorylation switch between the Linker and C-terminal plays crucial role in tumor progression and tumor cell apoptosis and that this switch may strictly be regulated by microRNA-145 expression in HCC. We found that expression of microRNA-21 and microRNA-145 was inversed and their roles in HCC may be strictly related to MAPK pathway activation and Smad3 phosphorylation at the Linker and C-terminal, the upstream of TGF-β signal. MicroRNA-21 and microRNA-145 are reported to be up-regulated and down-regulated respectively in liver disease as reviewed elsewhere [35], however, how these functionally opposing microRNAs interact with Smad3 in HCC remains veiled. MicroRNA-21 has exhaustively been shown to modulate Smad2/3 in many cell types [32, 41, 42] and promotes the proliferation and inhibits apoptosis in Eca109 cells via activating ERK1/2/MAPK pathway [43]. Although, microRNA-145 has severally been shown to modulate Smad3 expression in some cell types including human chondrocytes [36] and in some human diseases such as cystic fibrosis [26], however, how microRNA-145 modulates Smad3 in HCC remains unresolved. To precisely identify a specific site on Smad3, that is targeted in HCC by microRNA-145, a thorough search on MicroRNA database coupled with use of computational methods (MiRanda, TargetScan, PicTar) pointed to a locus on Smad3 gene which codes for proteins involved in immunosuppression and anti-inflammatory responses mediated by TGF-β [37]. Two putative microRNA-145 seed sites on 3’-UTR of Smad3 have been speculated. One (1397-1404 bp of NM_005902) of the two seed sites is shown to be active [26, 36]. Luciferase reporter construct containing Smad3 3’-UTR (pEZX-Smad3) and mutant Smad3 3’-UTR (pEZX-Smad3-mut) respectively, after co-transfection with microRNA-145 agomir in HepG2 cells led to significant decrease in luciferase activity (Figure 1). Implicit in this observation, is that microRNA-145 down-regulates Smad3 in HepG2 cells, but how this relates to phosphorylation of Smad3 at the Linker and the C-terminal is necessary, in view of the fact that domain-specific phosphorylation of Smad3 regulates different signals. For instance, pSmad3L mediates oncogenic signals as oppose to tumor suppressor effects of pSmad3C in HCC [44]. Analysis of xenograft tumors initially treated with microRNA-21 antagomir and 145 agomir produced inverse expression of microRNA-21 and microRNA-145. Importantly, up-regulated miR-145 was correlated with decreased pSmad3L and increased pSmad3C in tumors initially treated with microRNA-145 agomir, indicating semblance of interaction between these two factors. Further, this pattern of observation was related to decreased tumor burden and increased tumor cell apoptosis in xenograft tumors treated initially with microRNA-145 agomir compared to its respective negative control. Little changes in pSmad3C/3L expression but tumor inhibiting effects could be observed in miR-21 antagomir-group compared to antagomirNC-group. Clearly, indicating that microRNA-145 interacts with pSmad3C to mediate decrease tumor burden and increase tumor cell apoptosis in HCC while microRNA-21 shows promote tumor progression via an unknown mechanism. But spatio-temporal interaction between microRNA-21 and 145 and Smad3 phosphorylation was not apparent. To resolve this, using Smad3 phospho-specific plasmids (Smad3 EPSM and Smad3 3S-A) which after transfection in HepG2 cells successfully produced phosphorylation of Smad3C (Smad3-EPSM) and Smad3L (Smad3-3S-A), interestingly, it was observed that increase in pSmad3C correlated with increased expression of microRNA-145 while increased pSmad3L correlated with increased microRNA-21 expression in HepG2 cells compared to control and this observation confirmed not only the *in vivo* results but also indicated that indeed Smad3 phosphorylation precedesmicroRNA-21 and 145 expression in HepG2 cells (Figure 7).

Previously, microRNA-21 biosynthesis was demonstrated to be regulated by TGF-β and bone morphogenetic protein (BMP) stimulation via R-Smad (Smad1, Smad2, and Smad3) binding interactions. Specifically, binding of Smad2 and Smad3 to pri-miR-21 was observed secondary to TGF-β stimulation while Smad1 binding to pri-miR-21 was related to BMP stimulation [32], emphasizing that indeed interaction between R-Smads and microRNA biosynthesis may be ligand-related. Phosphorylation of Smad3 at the C-terminal is directly linked to ligand activation of TβRII and subsequent trans-phosphorylation of TβRI [31]; while Smad3 phosphorylation at the Linker is specifically the preserve of MAPK (ERK, JNK, and p38) activation secondary to TGF-β stimulation [31]. Therefore TβRI and TGF-β-mediated activation of MAPK are indispensable for Linker-specific phosphorylation of Smad3 [31]. To ascertain whether microRNA-21 and microRNA-145 expressions relate to these two rate-limiting steps in TGF-β signal, it was observed that inhibition of TβRI and MAPK pathway using TβRI-specific inhibitor (SB431542) and MAPK-specific inhibitors (PD98059, SP600125, and SB203580) in HepG2 cells produced increased expression of microRNA-145 but decreased expression of microRNA-21 (Figure 7) and this observation correspondingly led to a decrease in pSmad3L but increase in pSmad3C (Figure 3). Deductively, switch in Smad3 phosphorylation at the Linker and C-terminal strictly interact with microRNA-145 and microRNA-21 and this may in part be related to TβRI and MAPK pathway.

Dysregulated TGF-β signaling in cancer triggers biosynthesis of many oncogenic microRNAs including microRNA-21 at the post-transcriptional level, which in turn down-regulate tumor suppressor gene networks including microRNA-145 to promote oncogenesis [15, 45]. Dysregulated TGF-β signaling and its interaction with micrRNA-21 and 145 expression in cancer [12, 23, 38], as corroborated by the present study presents TGF-β and its signaling mediators, especially Smad3 as an important target for cancer therapy. Essentially, small molecules that can increase pSmad3C or decrease pSmad3L as well as global Smad3 expression may be useful for therapy. Also, small molecules of natural origin that can silence microRNA-21 but enhance microRNA-145 expression in HCC may be therapeutically useful for HCC treatment. But interaction between Smad3 phosphorylation and microRNA-145 biosynthesis, especially at the pre-transcriptional stage (pre-and-pri microRNA-145) needs to be investigated for possible targets.

To conclude, our evidence may be limited though, but preliminarily microRNA-21 increases tumor burden and decrease tumor cell apoptosis in a MAPK-dependent manner and microRNA-145 suppresses tumor progression via domain-specific phosphorylation of Smad3 in HCC. On the other hand, increased pSmad3C leads to microRNA-145 expression up-regulated and miR-21 down-regulated to restrain tumor progression while increased pSmad3L expression shows the opposite effect in HCC. The interaction between microRNA-21/145 and Smad3 domain-specific phosphorylation may regulate HCC progression, and the shift of pSmad3L to pSmad3C maybe an important target for HCC therapy.

## MATERIALS AND METHODS

### Animal models

SPF grade male BALB/c nude mice (14-18 g), aged 5 weeks were purchased from Vital River Laboratory Animal Technology Company (Beijing, China). The mice were kept in super clean laminar flow cabinet under the SPF conditions at the Experimental Animal Center of Anhui Medical University (Anhui, China). The mice were maintained under these conditions for 1 week for acclimatization before the commencement of experiments. The handling and use of the mice were carried out in accordance with the guidelines for the humane treatment of animals as set out by the Association of Laboratory Animal Sciences and the Center for Laboratory Animal Sciences at the Anhui Medical University.

### Grouping and treatment of animals

BALB/c nude mice were divided into four groups of six mice each as follows: miR-21 antagomirNC, miR-21 antagomir, miR-145 agomirNC and miR-145 agomir groups. After adaptation under SPF conditions for 1 week, animals were injected subcutaneously with HepG2 cell suspension (9×10^6^) to establish xenograft tumors as previously described [46]. After tumor formation, miR-21 antagomir (20 µl) (UCAACAUCAGUCUGAUAAGCUA) (GenePharma, Shanghai, China) and miR-21 antagomirNC (20 µl) (CAGUACUUUUGUGUAGUACAA) (GenePharma, Shanghai, China), miR-145 agomir (20 µl) (sense: GUCCAGUUUUCCCAGGAAUCCCU; antisense: GGAUUCCUGGGAAAACUGGACUU) (GenePharma, Shanghai, China), miR-145 agomirNC (20 µl) (sense: UUCUCCGAACGUGUCACGUTT; antisense: ACGUGACACGUUCGGAGAATT) (GenePharma, Shanghai, China) were respectively injected into xenograft every four days, for a total of 28 days as previously described [46]. In a separate experiment, eighteen BALB/c nude mice were divided into three groups of six mice each: Smad3-WT, Smad3-EPSM and Smad3-3S-A groups. After successful transfection of HepG2 cells respectively with Smad3-WT, Smad3-EPSM, and Smad3-3S-A plasmid, they were respectively injected into nude mice in each group. Successful transfection of HepG2 cells with plasmid was confirmed by immunofluorescence and western-blot. Animals were kept under strict observation throughout the experiment. Tumor tissues of each mice was harvested and stored at a temperature of -80 ^°^C until use.

### Cell culture

The human HCC HepG2 cell line was purchased from the Chinese Academy of Sciences Cell Bank (Shanghai, China). HepG2 cell lines were cultured in Dulbecco’s modified Eagle medium (DMEM) supplemented with 10 % fetal bovine serum (FBS) as previously described [47]. Cell cultures were maintained and incubated at 37 ^°^C in humidified air with 5 % CO_2_. The HepG2 cells with conventional culture were seeded at a density of 5×10^5^ on six well plates.

### Transfection of HepG2 Cells with Smad3 WT, Smad3 EPSM, and Smad3 3S-A

HepG2 cells were starved overnight. A 4 µl each of Lipofectamine^R^ LTX and Plus Reagent (Invitrogen, Shanghai, China) were mixed with opti-MEM (250 µl), 4 µl plasmid each of Smad3-WT, Smad3-EPSM, and Smad3-3S-A respectively (a gift from Dr Koichi Matsuzaki, kansai Medical University, Japan) and 4 µl plus. HepG2 cells were grown at 60-70 % confluence on 6 well plates, and then incubated with media for 6 h at 37 ^°^C and 5 % CO_2_ as previously described [47]. Non-transfected HepG2 cells were used as control. The medium containing Lipofectamine^R^ was removed, and the cells were incubated in DMEM medium with 10 % FBS. The cells were selected with G418 (600 µg/ml) (Sigma, Shanghai, China) 24h after transfection. Amplification of survived cells was used for experiments.

### Transfection of HepG2 Cells with microRNA-145 agomir and microRNA-21 antagomir

A 9 µl Lipofectamine^R^ RNAiMAX Transfection Reagent (Invitrogen, Shanghai, China) was mixed with opti-MEM (150 µl), opti-MEM (150 µl) containing miR-145 agomir (1.5 µl) or miR-21 antagomir (1.5 µl) (GenePharma, Shanghai, China), incubated for 5 min and then DMEM (1.7 ml) was added. After 6 h, the medium was removed with DMEM medium with 10 % FBS. 48 h after transfection, TGF-β_1_ (40 p mol) was added to activate the TGF-β/Smad signaling pathway.

### Luciferase Reporter Assay

MicroRNA-145 agomir and its negative control were purchased from GenePharma (Shanghai, China). The plasmid of 3’-UTR region of Smad3 (1397-1404bp of NM_005902) containing the predicted target sites of miR-145 (pEZX-Smad3) and the mutated seed sites within miR-145 (pEZX-Smad3 Mut) were purchased from GeneCopoeia (Guangzhou, China). All plasmid were DNA sequence confirmed before being used in our *in vitro* experiments. After that, we performed luciferase assay using these plasmids. The plasmid of pEZX-Smad3 Mut was used as control. HepG2 cells (4-5×10^4^) in 96-well plates were co-transfected with 100 ng of luciferase reporter constructs (pEZX-Smad3-WT or pEZX-Smad3-Mut), and 5 pmol of microRNA-145 agomir or miR-145 Negative Control with 0.5 µl Lipofectamine 2000 (Invitrogen) in 96-well plate. After Luciferase assays were performed 48 h after transfection using the Dual Luciferase Reporter Assay System (GeneCopoeia) according to the manufacturer’s protocol. Renilla/Firefly luciferase activity was determined for each reaction. Transfections and luciferase assays were carried out in triplicates and repeated three times.

### Immunofluorescence analysis

To detect the effect of stable transfection on intracellular localization of Smad3, the HepG2 cells and transfected cells were seeded at a density of 5×10^6^ /L on slides in a 24-well plate and then treated under the indicated conditions. The cells were fixed with 4 % paraformaldehyde, permeabilized with 0.1 % saponin, and blocked with 0.5 % bovine serum albumin, then incubated with primary antibody overnight at 4 ^°^C. The cells were incubated with fluorescein isothiocyanate (FITC)-conjugated secondary antibody for 2 h at room temperature, washed 3 times with phosphate buffer saline (PBS) for 5 min each time, incubated with 4’, 6-diamidino-2-phenylindole (DAPI) for 10 min at room temperature for nuclear staining. Finally, slides were mounted with 80 % phosphoglycerol, viewed and photographed using a fluorescent microscope (Olympus, Japan). Primary antibodies used in this study included anti-pSmad3C and anti-pSmad3L. At least 100 stained cells were analyzed per sample in each experiment.

### Western-blot analysis

Total proteins from cells and tissues were extracted by using Western blot and IP cell lysis liquid (Beyotime, Shanghai, China) according to standard procedures. Proteins were separated by sodium dodecyl sulfate/polyacrylamide gel electrophoresis (SDS/PAGE), transferred onto polyvinylidence difluoride (PVDF) membranes (Millipore, USA) by wet transfer method, blocked in 5 % skim milk powder dissolved in Tris-buffered Saline solution/0.1 Tween20 (TBST), incubated with the primary antibody overnight at 4 ^°^C, the next day, incubated with corresponding secondary antibody for 1h at room temperature, and finally the membranes were developed by using SuperSignalTM West Femto Trial Kit (Thermo Fisher Scientific, China) [37]. Primary antibody used in this study included anti-GAPDH (1:5000, Cell Signaling Technology, USA), anti-pSmad3C (1:1000, Cell signaling technology, USA), anti-pSmad3L (1:500, gift from Dr Koichi Matsuzaki, kansai Medical University, Japan), anti-Smad3 (1:500, Santa Cruz Biotechnology, China), anti-Smad4 (1:500, Santa Cruz Biotechnology, China), anti-JNK1/2 (1:1000, Cell signaling technology, USA), anti-pJNK1/2 (1:1000, Cell signaling technology, USA), anti-ERK (1:1000, Cell signaling technology, USA), anti-pERK (1:1000, Cell signaling technology, USA), anti-p38 (1:1000, Cell signaling technology, USA), anti-pp38 (1:1000, Cell signaling technology, USA).

### RNA isolation and quantitative real-time PCR (qRT-PCR) analysis

Total RNA was isolated from cells and tissues using TRIzol total RNA extraction reagent (Takara Biotechology, Dalian, China) according to protocol recommended by the manufacturer and were treated with RNase/DNase free. The concentration and purity of RNA were determined by measuring the absorbance at A260 and A260-A280 respectively. Total RNA (1 µg) was reverse transcribed using All-in-One™ miRNA qRT-PCR Detection Kit (Genecopoeia, Guangzhou, China). The reaction mixture was incubated at 37 ^°^C for 60min, inactivated at 85 ^°^C for 5min to inactivate the enzyme. qPCR was performed using the All-in-OneTM miRNA qRT-PCR Primer and SYBR Green I (Genecopoeia, Guangzhou, China). The qPCR reaction is performed with standard 3-step method, initial denaturation at 95 ^°^C for 10min, denaturation at 95 ^°^C for 10sec, annealing at 60 ^°^C for 20sec and extension at 72 ^°^C for 30sec (Bio-Red). The melting curve was analysis with ABI 7500 system to exclude contamination with unspecific PCR products. Relative expressions of target genes were determined by the 2^–ΔΔCt^ method [38]. The primers were as follows: hsa-miR21-5p (HmiRQP0316, Genecopoeia); hsa-miR-145-5p (HmiRQP0192, Genecopoeia); RNU6-2 (HmiRQP9001, Genecopoeia); rno-miR-21-5p (RmiRQP0316, Genecopoeia); rno-miR-145-5p (RmiRQP0192, Genecopoeia); snRNA U6 (RmiRQP9003, Genecopoeia).

### Hematoxylin and Eosin (H&E) Staining

Paraffin sections were de-paraffinized in xylene, rehydrated in series concentration alcohol, Immersed in distilled water for 30 sec. Sections were dipped in hematoxylin solution and agitated for 30 sec and then rinsed in distilled water for 1min. subsequently, sections were stained with 1 % eosin solution for 10-30 sec with agitation. Stained sections were dehydrated with increasing strength of alcohol (70 % and 90 % alcohol respectively) 10 min in each case, and then immersed in eosin for 3 min. The sections were dehydrated with alcohol and immersed in xylene. Stained sections were observed using electron microscope (Olympus, Japan).

### Immunohistochemical analyses

Xenograft tumor samples were preserved in 10 % paraformaldehyde solution, dehydrated and embedded in paraffin following routine methods. Briefly, paraffin sections were de-paraffinized in xylene and rehydrated in ethanol. Antigen retrieval was done by heating the sections to 121 ^°^C in 0.01 mol/L sodium citrate buffer (pH 6.0) for 10 min. After natural cooling, the sections were washed with PBS and incubated in 3 % H_2_O_2_ for 10 min to quench endogenous peroxidase activity. After washed with PBS, then Incubated with blocking buffer (normal goat serum) at room temperature for 20 min. the sections were incubated with primary antibodies over night at 4 ^°^C. Primary antibodies used in this study included anti-pSmad3C (1:100, Cell signaling technology, USA) and anti-pSmad3L (1:50, a gift from Dr K. Matsuzaki, kansai Medical University, Japan), anti-Smad4 (1:100, Santa Cruz Biotechnology, China). The sections were rewarmed at 37 ^°^C for 40 min, then washed with PBS and incubated with biotin-labeled the goat anti-mouse/rabbit lgG at 37 ^°^C for 30 min. Rinsing in PBS, and then the sections were incubated in HRP-labeled Streptavidin at 37 ^°^C for 30 min. Finally, the sections were developed with 3, 3’-diaminobenzidine counterstained with hematoxylin, cover slipped and evaluated using ImageJ software (NIH, Bethesda, MD, USA).

### Transmission electron microscopy analysis

The tumor tissues collected were sliced into 1 mm × 1 mm × 1 mm, immersed in 2.5 % Glutaraldehyde fixative solution for 4h at 4 ^°^C. Then the tissues were washed with 0.1 mol/L PBS at pH 7.2. Subsequently the tissues were fixed in 1 % osmic tetroxide for 2 h at room temperature and washed with PBS. Then the specimens were dehydrated in a gradient ethanol and acetone series. The specimens were embedded in embedding medium which is epoxy resin at 45 ^°^C for 2 h to remove the dehydrating agent. Subsequently, the tissues were embedded in embedding template, stained with lithium acetate, cut serially into 50 nm thickness using an ultra-microtome. The sections were observed by using a transmission electron microscope (JEM-1203, Japan).

### Statistical analysis

All statistical analysis was performed by using SPSS17.0 software (Chicago, IL). Data were expressed as mean ± standard deviation (SD). Multiple comparisons of means were analysis by one-way ANOVA. *P* < 0.05 was considered statistically significant.

## Abbreviations

HCC: hepatocellular carcinoma
pSmad3C and pSmad3L: Smad3 C-ternimal and Link region phosphorylation
TGF-β: transforming growth factor-beta
MAPK: mitogen-activated protein kinase
TβRI and TβRII: TGF-β type 1 and 2 receptors
SBE: Smad binding elements
PDCD4: programmed cell death 4 gene
HepG2 cells: humanized hepatoma cell lines
DMEM: Dulbecco’s modified Eagle medium
FBS: fetal bovine serum
SDS/PAGE: sodium dodecyl sulfate/polyacrylamide gel electrophoresis
PVDF: transferred onto polyvinylidence difluoride
TBST: Tris-buffered Saline solution/0.1 Tween20
FITC: fluorescein isothiocyanate
PBS: phosphate buffer saline
DAPI: 4’,6-diamidino-2-phenylindole
qRT-PCR: quantitative real-time polymerase chain reaction
BMP: bone morphogenetic protein
